# Geographical heterogeneity in prevalence of subclinical malaria infections at sentinel endemic sites of Myanmar

**DOI:** 10.1186/s13071-019-3330-1

**Published:** 2019-02-18

**Authors:** Ziling Liu, Than Naing Soe, Yan Zhao, Aye Than, Cho Cho, Pyae Linn Aung, Yuling Li, Lin Wang, Huilin Yang, Xiangnan Li, Danni Li, Zhiping Peng, Jiangang Wang, Yan Li, Zhaoqing Yang, Hongning Zhou, Qinghui Wang, Myat Phone Kyaw, Yaming Cao, Liwang Cui

**Affiliations:** 1 Department of Immunology, College of Basic Medical Sciences, China Medical University, Shenyang, 110122 Liaoning China; 2 Department of Clinical Laboratory, Baotou central Hospital, Baotou, 014040 Inner Mongolia Province China; 3 Department of Public Health, Ministry of Health and Sports, Nay Pyi Taw, Myanmar; 4 Myanmar Medical Association, Yangon, Myanmar; 5 Department of Pathogen Biology and Immunology, Kunming Medical University, Kunming, 650000 China; 6 Yunnan Provincial Center of Arborvirus Research, Yunnan Provincial Key Laboratory of Vector-borne Diseases Control and Research, Yunnan Institute of Parasitic Diseases, Puʼer, 665000 Yunnan China; 7 Department of Internal Medicine, Morsani College of Medicine, University of South Florida, 3720 Spectrum Boulevard, Suite 304, Tampa, FL 33612 USA

**Keywords:** Myanmar, Malaria, Prevalence, Subclinical, Pooling strategy

## Abstract

**Background:**

The malaria burden of Myanmar still remains high within the Greater Mekong Subregion of Southeast Asia. An important indicator of progress towards malaria elimination is the prevalence of parasite infections in endemic populations. Information about malaria epidemiology is mostly derived from reports of confirmed acute malaria cases through passive case detection, whereas the prevalence of baseline subclinical malaria infections is much less known.

**Methods:**

In this study, cross-sectional surveys were conducted during the rainy season of 2017 in four townships (Bilin, Thabeikkyin, Banmauk and Paletwa) of Myanmar with divergent annual malaria incidences. A total of 1991 volunteers were recruited from local villages and *Plasmodium* subclinical infections were estimated by light microscopy (LM), rapid diagnostic tests (RDTs) and nested PCR. The nested PCR analysis was performed with a modified pooling strategy that was optimized based on an initial estimate the infection prevalence.

**Results:**

The overall malaria infection prevalence based on all methods was 13.9% (277/1991) and it differed drastically among the townships, with Paletwa in the western border having the highest infection rate (22.9%) and Thabeikkyin in central Myanmar having the lowest (3.9%). As expected, nested PCR was the most sensitive and identified 226 (11.4%) individuals with parasite infections. Among the parasite species, *Plasmodium vivax* was the most prevalent in all locations, while *Plasmodium falciparum* also accounted for 32% of infections in the western township Paletwa. Two RDTs based on the detection of the hrp2 antigen detected a total of 103 *P. falciparum* infections, and the ultrasensitive RDT detected 20% more *P. falciparum* infections than the conventional RDT. In contrast, LM missed the majority of the subclinical infections and only identified 14 *Plasmodium* infections.

**Conclusions:**

Cross-sectional surveys identified considerable levels of asymptomatic *Plasmodium* infections in endemic populations of Myanmar with *P. vivax* becoming the predominant parasite species. Geographical heterogeneity of subclinical infections calls for active surveillance of parasite infections in endemic areas. The pooling scheme designed for nested PCR analysis offers a more practical strategy for large-scale epidemiological studies of parasite prevalence. Such information is important for decision-makers to put forward a more realistic action plan for malaria elimination.

**Electronic supplementary material:**

The online version of this article (10.1186/s13071-019-3330-1) contains supplementary material, which is available to authorized users.

## Background

Globally, malaria remains a substantial public health problem and significant economic burden with an estimated 219 million cases and 435,000 deaths in 2017 [1]. Malaria cases and deaths in Southeast Asia accounted for 7 and 6% of the global burden, respectively. In Southeast Asia, the six countries within the Greater Mekong Subregion (GMS) have a regional goal of malaria elimination by 2030. To achieve this goal, control efforts to continuously shrink the malarious areas have intensified, whereas in areas where malaria transmission had been interrupted, increased surveillance has been implemented to maintain the malaria-free status and prevent malaria re-introduction [2]. According to the World Malaria Report 2018 [1], the Myanmar malaria burden still remains high within the GMS, and malaria transmission persists in most international border regions. This poses a serious threat to the neighboring countries and greatly hinders regional malaria elimination. Compared to acute malaria cases, asymptomatic malaria infections constitute silent reservoirs of transmission [3]. Therefore, universal coverage of human populations with early diagnosis and effective treatment is essential to reduce morbidity, mortality and transmission. Case detection can be done through passive case detection in hospitals and clinics and active case detection by regular house visits, whereas cross-sectional surveys are conducted to determine the prevalence of infections (mostly asymptomatic). Reports for confirmed malaria cases have shown drastic heterogeneity in malaria distribution, which may also correspond to the prevalence of asymptomatic infections [4–6]. Typically, most malaria infections in endemic areas are asymptomatic, while confirmed cases of acute malaria are just “the tip of an iceberg”. Since subclinical infections and complicated transmission patterns can contribute to the persistence of malaria, a reliable estimation of the prevalence of asymptomatic malaria is important to guide and monitor progress toward control and elimination [7].

Conventionally, malaria diagnosis is done by light microscopy (LM), which normally has a detection limit of 10–20 parasites/μl blood in thick film and 100 parasites/μl blood in thin film [8, 9]. In addition, misdiagnosis of the parasite species is common and depends to a large extent on the experience of the microscopist [8, 10]. Rapid diagnostic tests (RDTs) for antigen detection, although not as technically demanding as LM, also have similar detection limits as LM. Given that a large proportion of subclinical malaria infections have parasite densities below the detectable limit of LM and RDTs, molecular detection methods are required for a more accurate estimate of the overall malaria infection rate. PCR-based methods are the most commonly used molecular assays, which can detect parasites to the genus- or species-specific level [11–14]. However, the use of PCR in epidemiological surveillance is mostly done on a small scale, since the procedure is both laborious and costly. For large-scale surveys, pooling strategies are recommended in order to reduce the cost and time [15–19].

This study aimed to assess malaria prevalence at sentinel sites in Myanmar and provide baseline information for the evaluation of progress toward malaria elimination. Cross sectional surveys were conducted in four townships with different levels of endemicity (from reports of confirmed malaria cases), and parasite infection rates were estimated by using LM, two types of RDTs, and nested PCR incorporating a sample pooling design.

## Methods

### Study sites and blood sample collection

**Fig. 1:**
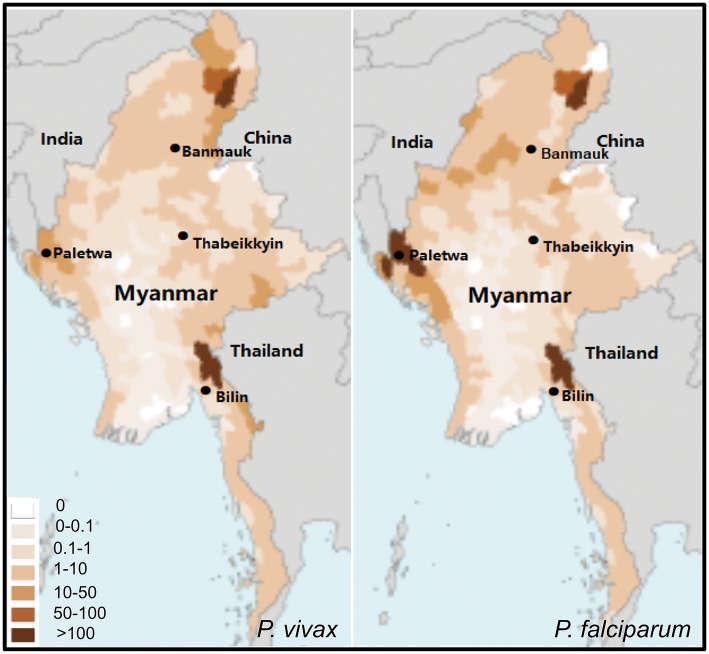
Locations of the four townships where the cross-sectional surveys were conducted. Maps show the distribution of confirmed *P. vivax* (left) and *P. falciparum* (right) cases in Myanmar in 2016. Annual malaria incidences (confirmed cases per 1000 population) are color-coded

Four Myanmar townships Paletwa (western), Banmauk (northern), Thabeikkyin (central) and Bilin (southeastern) with different malaria transmission intensities were selected to conduct cross-sectional surveys to assess malaria prevalence during the rainy season of 2017 (Fig. 1). The 2016 confirmed malaria cases show that Paletwa had the highest annual incidence rate of malaria (> 100‰ *P. falciparum* and 10–50‰ *P. vivax*), followed by Banmauk (1–10‰ *P. falciparum* and *P. vivax*), Bilin (1–10‰ *P. falciparum* and *P. vivax*) and Thabeikkyin (0.1–1‰ *P. vivax* and *P. falciparum*) [20]. Participants were interviewed and information on gender, age, and axillary temperature was recorded. Those with a temperature of 37.3 °C or above were considered febrile. After consent, finger-prick blood was collected for malaria diagnosis by light microscopy (LM), rapid diagnostic tests (RDTs), and spotted onto filter paper for PCR analysis.

### Malaria diagnosis by LM and RDTs

For LM diagnosis of malaria, Giemsa-stained blood smears were read by microscopists at the field sites following an established standard operating procedure [21]. A slide was considered positive when at least one parasite was found [22, 23]. Two RDTs, SD Bioline malaria Ag *Pf* test (Alere, Yongin-si, Republic of Korea), a conventional RDT (cRDT); and Alere™ malaria Ag *Pf* RDT (Alere), an ultrasensitive RDT (uRDT); were employed to detect *P. falciparum* infections. Both RDTs were based on the detection of *P. falciparum* HRP-2 antigen.

### Malaria diagnosis by nested PCR

To detect parasite DNA, genomic DNA (gDNA) was isolated from the dried blood spots on filter paper using a QIAmp DNA Mini Kit (Qiagen, Hilden, Germany). Isolated genomic DNA was eluted into 35 μl of elution buffer and used immediately or stored at -20 °C until further use. For parasite detection, genus-specific nested PCR and species-specific nested PCR assays were performed using 2 μl of gDNA and primers (see Additional file 1: Table S1) as described previously [8, 11, 24, 25].

### Sample pooling design

**Fig. 2:**
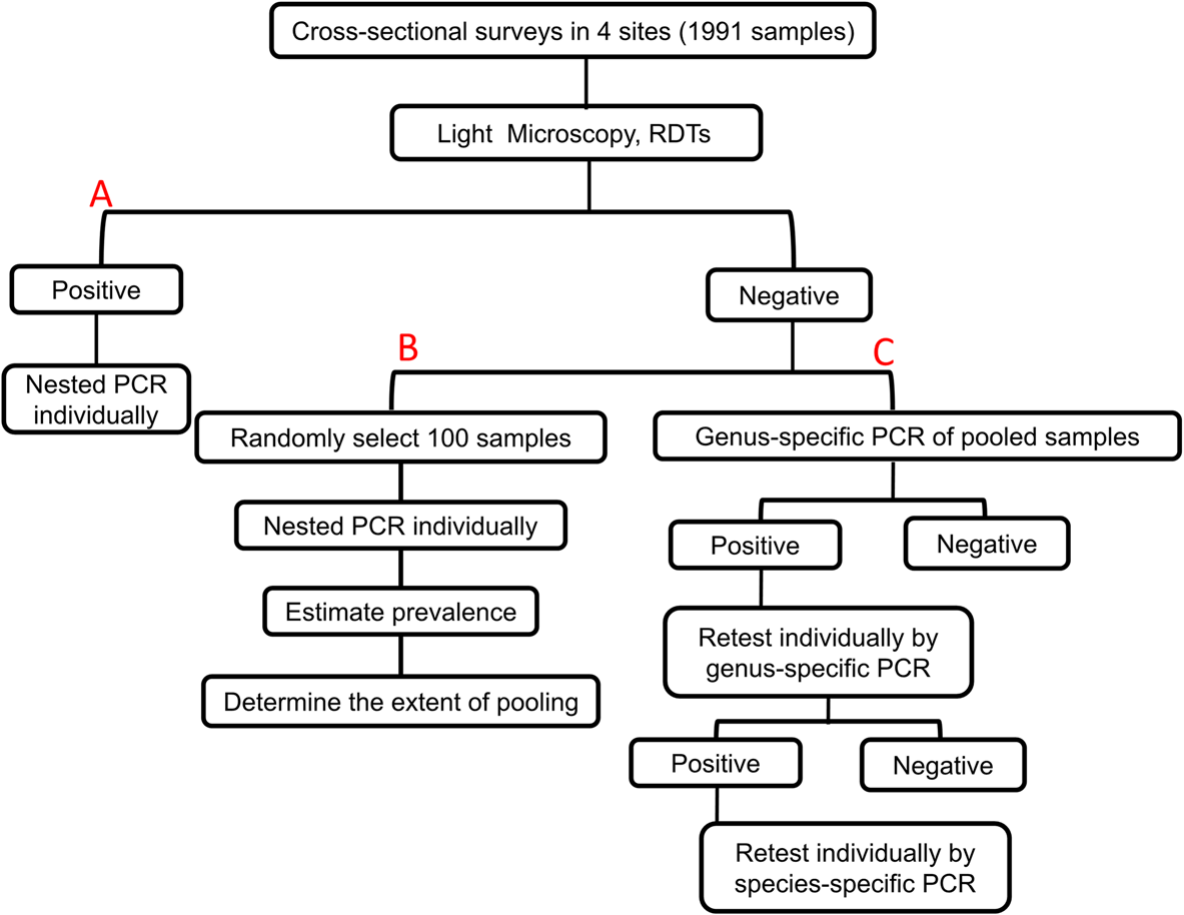
Sample processing and assay work flow of the nested PCR pooling design. A: All samples positive by LM and RDTs were validated individually by nested PCR. B: Initial analysis of 100 random samples from the negatives by LM and RDT to estimate the infection prevalence and determine the degree of pooling. C: Pooling design of nested PCR of the remaining negative samples by LM and RDT

For each positive sample identified by LM and/or RDT, species-specific nested PCR was performed to confirm the results. For the remaining negative samples, a pooling strategy was designed [19]. First, a small random set of 100 samples was selected and parasite prevalence was evaluated individually by nested PCR using *Plasmodium* genus-specific primers of rPLU1/rPLU5 (Nest1) and rPLU3/rPLU4 (Nest2). To design a pooling strategy for the remaining negative samples, a contingency table (Table 1) was generated to obtain an expected percent reduction of workload, R_save_, for infection rate using the following formula: R_save_ = T_save_/T_individual_ ×100% = (2+P×n_1_–2/N–2PN–P×n2)/(2+P×n_1_), T_individual_ = S×2+P×S×n_1_=S×(2+P×n_1_), T_pooling_ = (S/N)×2+P×S×N×2+P×S×n2=S×(2/N+2P×N+P×n2), T_save_ = T_individual_ – T_pooling=_ S×(2+P×n_1_) –S×(2/N+2PN+P×n2) =S×(2+P×n_1_–2/N–2PN–P×n2), where T_individual_ refers to the number of tests performed if the samples are tested individually, T_pooling_ the number of tests performed if the samples are pooled, and T_save_ the number of tests saved if the samples are pooled *versus* tested individually. In the formula, P is the estimated infection rate, N number of samples in each pool, n_1_ number of species (5 in this study), and n_2_ the number of groups of the species are divided into for nested PCR (3 in this study). R_save_ was estimated assuming that each pool contains no more than one positive sample. For each positive pool, samples were first tested individually by using genus-specific nested PCR (Fig. 2). For the positive samples, species-specific primers for *P. falciparum*, *P. vivax*, *P*. *malariae*, *P. ovale* and *P*. *knowlesi* were used to identify the *Plasmodium* species using primary PCR product as the template. Nested PCR for species were grouped into three reactions and multiplexed for *P. falciparum* + *P*. *knowlesi* and *P. malariae* + *P. ovale* based on easy separation of the PCR products. PCR products were separated in 1.2% agarose gel for visualization.

**Table 1:** Percent reduction (%) of workload for different extent of sample pooling (2–8 samples/pool) based on estimated infection rates (1–20%)

Estimated infection rate (%)	Number of samples per pool						
	2	3	4	5	6	7	8
1	47.80	63.09	70.24	74.15	75.93	77.77	78.54
2	45.71	59.68	65.71	68.57	69.37	70.20	70.00
3	43.72	56.43	61.40	63.26	63.10	62.99	61.86
4	41.82	53.33	57.27	58.18	57.12	56.10	54.09
5	40.00	50.37	53.33	53.33	51.41	49.52	46.67
6	38.26	47.54	49.57	48.70	45.94	43.23	39.57
7	36.60	44.82	45.96	44.26	40.71	37.20	32.77
8	35.00	42.22	42.50	40.00	35.69	31.43	26.25
9	33.47	39.73	39.18	35.92	30.88	25.89	20.00
10	32.00	37.33	36.00	32.00	26.27	20.57	14.00
11	30.59	35.03	32.94	28.24	21.83	15.46	8.24
12	29.23	32.82	30.00	24.62	17.56	10.55	2.69
13	27.92	30.69	27.17	21.13	13.46	5.82	-2.64
14	26.67	28.64	24.44	17.78	9.51	1.27	-7.78
15	25.45	26.67	21.82	14.55	5.70	-3.12	-12.73
16	24.29	24.76	19.29	11.43	2.02	-7.35	-17.50
17	23.16	22.92	16.84	8.42	-1.52	-11.43	-22.11
18	22.07	21.15	14.48	5.52	-4.94	-15.37	-26.55
19	21.02	19.44	12.20	2.71	-8.25	-19.18	-30.85
20	20.00	17.78	10.00	0.00	-11.44	-22.86	-35.00

### Statistical analysis

Data were analyzed by SPSS v.22.0. Malaria prevalence was calculated by crosstabs. The odds ratio (OR) was calculated for binary variables and Chi-square or Fisher’s exact tests were used where appropriate. *P* < 0.05 was considered statistically significant. The sensitivity and specificity of uRDT and cRDT in detecting slide-positive and nested PCR-positive *P. falciparum* infections in our study population were calculated by crosstabs with LM or nested PCR as the gold standard.

## Results

### Geographical variations in the prevalence of malaria infections

**Fig. 3:**
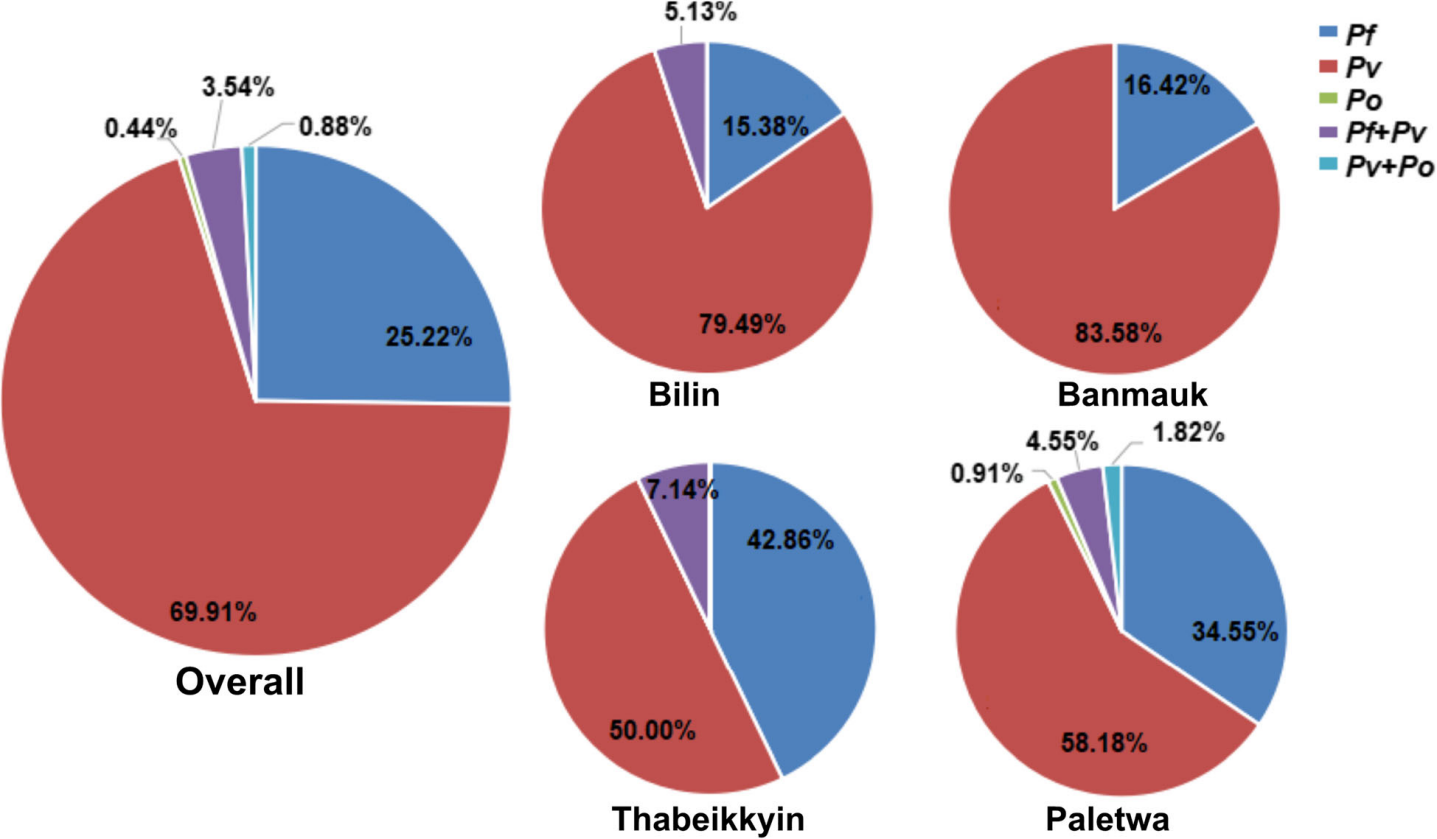
Malaria species compositions in different study sites. The pie graphs showed the percentage of each species identified in four study areas and all populations. *Abbreviations*: *Pf*, *P. falciparum*; *Pv*, *P. vivax*; *Po*, *P. ovale*

A total of 1991 participants were enrolled for the cross-sectional surveys of malaria prevalence: Bilin (*n* = 315), Banmauk (*n* = 631), Thabeikkyin (*n* = 414) and Paletwa (*n* = 633). The overall parasite prevalence was 13.9% (277/1991) when all positive samples identified by at least one method were considered (Table 2). The limits of detection of the nested PCR was 0.1–1, 0.1–1, 1–5 and 1–5 parasites/μl for *P. falciparum*, *P. vivax*, *P. malariae* and *P. ovale*, respectively [8]. As expected, nested PCR was the most sensitive and detected 226 (11.4%) *Plasmodium* infections in the study populations. In stark contrast, LM only detected 13 *P. falciparum* and one for *P. vivax*, probably reflecting the low parasite densities associated with asymptomatic infections in low-endemicity regions. Screening by *P. falciparum* infections by cRDTs and uRDTs estimated parasite prevalences of 3.7% (73/1991) and 5.0% (100/1991), respectively. For parasite species, *P. vivax* accounted for the majority of the infections 69.9% (158/226), while *P. falciparum* and *P. ovale* were responsible for 25.2% (57/226) and 0.4% (1/226), respectively. Mixed infections by *P. vivax/P. falciparum* and *P. vivax/P. ovale* accounted for 3.5% (8/226) and 0.9% (2/226), respectively (Fig. 3). Neither *P. knowlesi* nor *P. malariae* was identified.

**Table 2:** Malaria prevalence [number (%)] in different townships

Townships	No. of participants surveyed	LM	cRDT	uRDT	LM, RDT, or both	Nested PCR						Total
						Pv	Pf	Po	Pv + Pf	Pv + Po	Total	
Bilin	315	0	0	0	0	31	6	0	2	0	39 (12.4)	39 (12.4)
Banmauk	631	3 (0.5)	5 (0.8)	16 (2.5)	18 (2.9)	56	11	0	0	0	67 (10.6)	77 (12.2)
Thabeikkyin	412	0 (0)	1 (0.2)	3 (0.7)	3 (0.7)	7	6	0	1	0	14 (3.4)	16 (3.9)
Paletwa	633	11 (1.7)	67 (10.6)	81 (12.8)	82 (13.0)	64	34	1	5	2	106 (16.7)	145 (22.9)
Total	1991	14 (0.7)	73 (3.7)	100 (5.0)	103 (5.2)	158	57	1	8	2	226 (11.4)	277 (13.9)

*Abbreviations*: *LM* light microscopy, *cRDT* conventional RDT, *uRDT* ultrasensitive RDT, *Pv P. vivax*, *Pf P. falciparum*, *Po P. ovale*

The four townships had significantly different prevalences of malaria infections and parasite species compositions. Consistent with the malaria endemicity from confirmed malaria cases, Paletwa had the highest rate (22.9%) of asymptomatic malaria infections, followed by Bilin and Banmauk (~12%), and Thabeikkyin had the lowest prevalence (3.9%). LM only identified parasite infections in the western and northern townships, whereas it did not detect any in the eastern and central townships. In the southeastern and northern townships, *P. vivax* was the predominant species detected, accounting for ~80% or higher proportions of infections, whereas the western township Paletwa still had a considerable proportion (~34%) of *P. falciparum* infections (Fig. 3). It is noteworthy that Bilin, considered malaria-free based on microscopy and RDTs, still had a substantial level (12.4%) of asymptomatic *vivax* infections (Table 2, Fig. 3).

### Comparison of different methods in detecting *P. falciparum* infections

**Fig. 4:**
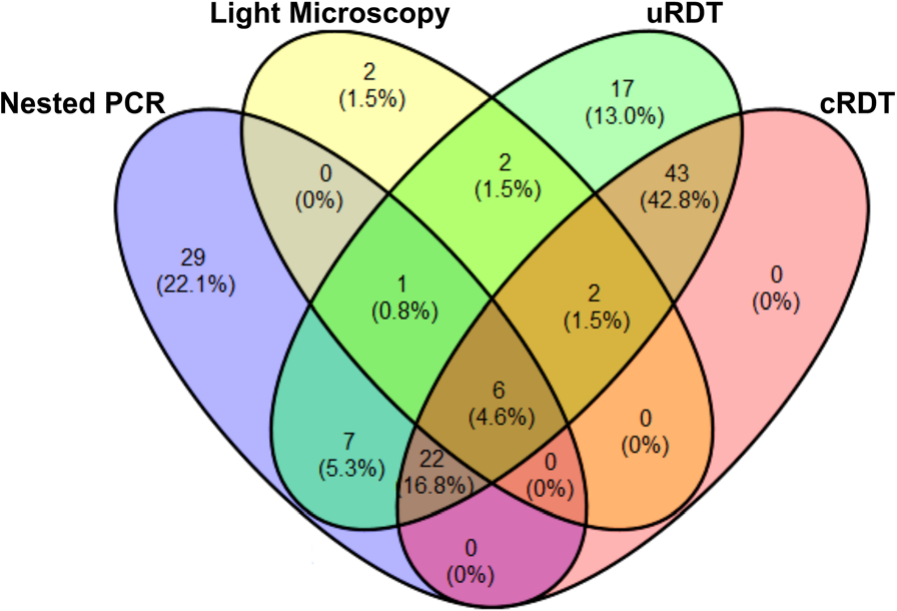
Venn diagram showing the overlap in the number (percentage) of individuals with *P. falciparum* infections in 1991 volunteers as detected by LM, nested PCR, uRDT and cRDT

Of the 1991 participants, 131 were diagnosed with *P. falciparum* infections by at least one method, of which 3.3% (*n* = 65), 0.7% (*n* = 13), 3.7% (*n* = 73) and 5.0% (*n* = 100) were detected by nested PCR, LM, cRDT and uRDT, respectively. A Venn diagram shows the overlap in the number (percent) of individuals with *P. falciparum* infections detected by the four methods (Fig. 4). Of all the positive samples, only six participants were diagnosed with *P. falciparum* by all methods. For the 13 *P. falciparum* positives determined by LM, 11, 8 and 7 were also detected by uRDT, cRDT and nested PCR, respectively, whereas two samples were failed by both nested PCR and RDTs. Of all 100 *P. falciparum* positives by uRDT, 17 samples were negative by all the other detection methods (nested PCR, LM and cRDT). uRDT detected all samples that were positive by cRDT. Among 65 *P. falciparum* positives from nested PCR, only 10.8% (*n* = 7), 55.4% (*n* = 36) and 43.1% (*n* = 28) were also positive by LM, uRDT and cRDT, respectively, whereas 44.6% (*n* = 29) was not detected by the rest of detection methods (LM, uRDT and cRDT).

The sensitivity and specificity of the two RDTs for detecting *P. falciparum* infections in the cross-sectional surveys were evaluated using LM or nested PCR as the gold standard (Table 3). Overall, the specificities of both RDTs were high (> 95%). When LM was used as reference, both RDTs showed modest sensitivities (84.6% and 61.5%). Their sensitivities were much lower (55.4% and 43.1%) when nested PCR was used as the gold standard. Clearly, uRDT had much higher sensitivities than cRDT and detected 20% more positive cases than cRDT.

**Table 3:** Sensitivity and specificity of the two RDTs for detecting parasite infections

Gold standard		uRDT				cRDT			
		Positive (*n*)	Negative (*n*)	SE (%) (95% CI)	SP (%) (95% CI)	Positive (*n*)	Negative (*n*)	SE (%) (95% CI)	SP (%) (95% CI)
LM	Positive	11	2	84.6 (61.9–100.0)	95.5 (94.6–96.4)	8	5	61.5 (30.9–92.1)	96.7 (95.9–97.5)
	Negative	89	1889			65	1923		
Nested PCR	Positive	36	29	55.4 (43.0–67.8)	96.9 (95.9–97.5)	28	37	43.1 (30.7–55.4)	97.7 (97.0–98.3)
	Negative	64	1862			45	1881		

*Abbreviations*: *SE* sensitivity, *SP* specificity, *95% CI* 95% confidence interval

### Risk factors of subclinical malaria infections

Because the visits to the surveyed villages were done in the daytime, when adults and men were probably away from home engaging in agricultural activities, the study population were slightly female-biased (56.91%) and relatively young (median age: 14 years, range: 7 months to 90 years) (Table 4). Parasite prevalence was similar in both male and female populations, and also similar among the three age groups examined. Of the 1991 participants, 29 individuals had fever at the time of the survey. Among them, 14 were infected with *Plasmodium*, but only five had a parasitemia that was high enough to be detected by LM and could be considered as having acute malaria episodes. By the binary logistic regression analysis, febrile participants had significantly higher prevalence and odds of malaria infection than the non-febrile (OR = 7.51, *P* < 0.001).

**Table 4:** Study population demographics and risk analysis

		*N* (%)	Prevalence, *n* (%)	OR (95%CI)	*P*-value
Sex	Female	1133 (56.9)	126 (11.1)	1	
	Male	858 (43.1)	105 (12.2)	1.11 (0.85–1.47)	0.441
Fever	No	1962 (98.5)	217 (11.1)	1	
	Yes	29 (1.5)	14 (48.3)	7.51 (3.57–15.76)	<0.0001
Age groups (years)	≤ 5	228 (11.5)	29 (12.7)	1.15 (0.74–1.78)	0.544
	6–14	814 (40.9)	95 (11.7)	1.04 (0.78–1.40)	0.801
	≥ 15	949 (47.7)	107 (11.3)	1	

*Abbreviations*: *OR* odds ratio, *CI* confidence interval

### Efficiency of the pooling strategy for molecular epidemiological studies

To obtain highly accurate infection prevalence by molecular methods in low-endemic areas, sample pooling is necessary. Table 1 shows the calculated reduction of workload under different extents of pooling based on an estimated infection rate of 1–20%. In a small set of 100 randomly selected negative samples by both LM and RDTs, nested PCR analysis identified 9% *Plasmodium*-positive samples. At this infection rate, having three samples per pool would give a maximum reduction of workload (~40%). Excluding 103 positive samples identified by LM and/or RDTs, 1788 samples were divided into 596 pools and genus-specific nested PCR identified 126 pools as positive. All samples from the 126 positive pools were amplified individually by genus-specific nested PCR (756 reactions), of which 158 were positive. To confirm parasite species, each of the 158 samples was re-tested in three reactions of species-specific PCR (total 474 reactions). With this pooling design, a total of 2170 PCR reactions were performed as compared to 4050 if all samples had been analyzed individually (1788 × 2 + 158 × 3). In another word, this pooling strategy reduced the workload by 46.4%.

## Discussion

It is believed that low-density asymptomatic infections are expected to be common in high-endemic areas with high herd immunity and premunition in the population [26]. Contrary to this conventional belief, asymptomatic infections are also found to be prevalent in areas of low endemicity [27]. A recent epidemiological study in Cambodia, Vietnam and along the Thailand-Myanmar border showed that approximately 20% of the population in these settings harbored *P. falciparum* or *P. vivax* infections, most of which were afebrile and asymptomatic [28]. Similarly, this report found 3.9–22.9% of the residents in four malaria endemic areas carrying *Plasmodium* infections. Compared with the confirmed acute malaria infections reported in 2017, the different prevalence of subclinical infections in these endemic areas appeared to correspond to the levels of acute malaria and very well reflected the local malaria transmission intensities. The majority of the asymptomatic infections were present at parasite densities below the detection limit of LM. Such submicroscopic infections could develop into much higher-density infections at a later time and cause acute diseases [29, 30]. In addition, they are capable of infecting mosquitoes and contributing to transmission [31–33]. Therefore, low-density asymptomatic malaria parasitemia could influence transmission dynamics and sustain malaria endemicity, which might undermine elimination efforts [34]. Asymptomatic infections need to be closely monitored and dealt with adequate control measures in regions pursuing malaria elimination.

This study identified significant heterogeneity in the prevalence of subclinical malaria infections in different geographical regions of Myanmar, a country with the heaviest malaria burden in the GMS. The prevalence of *Plasmodium* infections in the four townships surveyed ranged from 3.9 to 22.9%. Paletwa, located in the south-west site of Chin State had the highest parasite prevalence. Its mountainous terrain and poor accessibility thwart malaria control efforts. Bordering malarious areas of Bangladesh and India further promotes travel-associated malaria and malaria introduction [35]. Interestingly, the study population in Bilin had no detectable parasite infections by LM and RDTs, but nested PCR analysis revealed a substantial level of submicroscopic infection. This geographical heterogeneity indicates that malaria control strategy needs to be tailored to specific regions with different malaria epidemiology. While proper case management continues to be a focus of control efforts, novel strategies are needed to target the silent parasite reservoirs.

This study assessed several available malaria diagnostic tools for screening asymptomatic malaria infections. LM examination, the traditional gold standard for diagnosis of clinical malaria, performed poorly in this study with only 14 *Plasmodium* infections detected, accounting for 5% (14/277) of total infections identified by other methods. This is likely due to the low parasite densities associated with the asymptomatic infections, especially by *P. vivax*. Therefore, the use of microscopy is likely to result in a gross underestimation of the malaria burden [36]. RDTs for detection of *Plasmodium* antigens are commonly used for malaria diagnosis due to its simplicity. Conventional RDTs have similar detection limits as microscopy, but uRDTs may be ten times more sensitive than cRDTs. This study showed that uRDTs identified 20% more *P. falciparum* infections. However, one limitation for the *P. falciparum* RDTs is the high prevalence of *P. vivax* infections in the GMS, and uRDTs to detect *P. vivax* are not available. Moreover, other disadvantages of HRP-2 based RDTs are the presence of *P. falciparum* parasites with *hrp2* gene deletion in the study region (cause of false negatives) [25], and continuous circulation of HRP-2 antigen long after successful treatment of infections (cause of false positives). The latter would exaggerate the rate of infections at the time of the surveys and is probably the major reason for the discrepancy in detection results between RDTs and nested PCR in our study. PCR is the method of choice for epidemiological studies of malaria, but the high cost of labor and reagents prevents its use for large-scale screening purposes. To make it more practical for large-scale epidemiological studies, a pooling strategy would be more cost effective [8, 18]. A pooling strategy is further developed in this study, where the degree of sample pooling is optimized based on estimated prevalence of infections, which can be determined by studying a small, random subset of samples. Given the changing malaria epidemiology this step is necessary, especially for areas where the prevalence is under 10% [37–39]. In this study, with an estimated parasite infection rate of 9%, the pooling nested PCR reduced the workload by 46.4%.

## Conclusions

This study shows that large proportions of individuals in malaria endemic areas of Myanmar carried subclinical malaria infections with pronounced geographical heterogeneity. In all four townships, *P. vivax* was the predominant species. In the western border township *P. falciparum* was also substantially prevalent. Whereas LM was deemed not suitable for detecting subclinical infections in these low-endemicity areas, the nested PCR method incorporating a pooling strategy would enable the implementation of molecular epidemiology studies on a large scale. Such information is important to decision-makers for future plans in the national malaria elimination program.

## Additional file

Additional file 1: **Table S1.** Primers for pooling strategy of nested PCR (*Plasmodium* spp. detection). (DOCX 15 kb)

## Abbreviations

CI: Confidence interval
cRDT: Conventional RDT
LM: Light microscopy
OR: Odds ratio
PCR: Polymerase chain reaction
RDTs: Rapid diagnostic tests
uRDT: Ultrasensitive RDT
