# Supplementary material for: Geographical heterogeneity in prevalence of subclinical malaria infections at sentinel endemic sites of Myanmar
Source: Parasit Vectors. 2019 Feb 18;12:83. doi: 10.1186/s13071-019-3330-1 (PMC6378722; doi:10.1186/s13071-019-3330-1)
Supplement: Supplementary file 1 — Table S1. Primers for pooling strategy of nested PCR (Plasmodium spp. detection). (DOCX 15 kb) [file 13071_2019_3330_MOESM1_ESM.docx]

**Additional file 1: Table S1** Primers for pooling strategy of nested PCR (*Plasmodium* spp. detection)

|  | **Primers for genus-specific** | **Primer sequences (5’ to 3’)** | **Amplicon Size（bp）** | **Primers for species-specific** | | **Primer sequences (5’ to 3’)** | **Amplicon Size（bp）** |
| --- | --- | --- | --- | --- | --- | --- | --- |
| **Nested 1** | rPLU1_F | TCA AAG ATT AAG CCA TGC AAG TGA | ≈1670 | rPLU1_F | | TCA AAG ATT AAG CCA TGC AAG TGA | ≈1670 |
|  | rPLU5_R | CCT GTT GTT GCC TTA AAC TTC |  | rPLU5_R | | CCT GTT GTT GCC TTA AAC TTC |  |
| **Nested 2** | rPLU3_F | TTT TTA TAA GGA TAA CTA CGG AAA AGC TGT | ≈240 | *P.f* | rFAL1 | TTAAACTGGTTTGGGAAAACCAAATATATT | ≈206 |
|  | rPLU4_R | TAC CCG TCA TAG CCA TGT TAG GCC AAT ACC |  |  | rFAL2 | ACACAATGAACTCAATCATGACTACCCGTC |  |
|  |  | | | *P.v* | PV18S F | GAATTTTCTCTTCGGAGTTTATTC | ≈419 |
|  |  |  |  |  | PV18S R | GTAGAAAAGGGAAAGGGAAACTGTTA |  |
|  |  |  |  | *P.m* | rMAL1 | ATAACATAGTTGTACGTTAAGAATAACCGC | ≈145 |
|  |  |  |  |  | rMAL2 | AAAATTCCCATGCATAAAAAATTATACAAA |  |
|  |  |  |  | *P.o* | rOVA1WC | TGTAGTATTCAAACGCAGT | ≈660 |
|  |  |  |  |  | rOVA2WC | TATGTACTTGTTAAGCCTTT |  |
|  |  |  |  | *P.k* | PK18SF | GAG TTT TTC TTT TCT CTC CGG AG | ≈424 |
|  |  |  |  |  | PK18SR | GGG AAA GGA ATC ACA TTT AAC GT |  |
